# RELIEF, REGRETS, AND REINVENTION: LIFE AFTER GRAY DIVORCE

**DOI:** 10.1093/geroni/igac059.158

**Published:** 2022-12-20

**Authors:** Jacquelyn Benson, Landon Olivia, Allison Donehower, Caroline Sanner

**Affiliations:** Washington University in St. Louis, Columbia, Missouri, United States; University of Missouri, Columbia, Missouri, United States; University of Missouri, Columbia, Missouri, United States; Virginia Tech, Blacksburg, Virginia, United States

## Abstract

“Gray divorce,” or divorce which occurs in later life, is rapidly becoming more common in the United States. The purpose of this grounded theory study was to examine the lived experience of getting divorced in mid to later life. Data address the following research questions: 1) What are the divorcees’ expectations for the process of divorce and post-divorce life? 2) How do life phase factors and family relationships shape the divorce experience? Participants included 41 heterosexual men and women who legally divorced at the age of 55 or older and between 1-7 years from the time of the interview. Participants divorced from first and higher order marital unions. They included self-identified initiators, non-initiators, and co-initiators of the divorce. Results suggest that gray divorce is a complex experience marked by shifting feelings of ambivalence and certainty that are influenced by such factors as health and social networks, especially family.

